# Risk of Gastric Cancer by Water Source: Evidence from the Golestan Case-Control Study

**DOI:** 10.1371/journal.pone.0128491

**Published:** 2015-05-29

**Authors:** Laura Eichelberger, Gwen Murphy, Arash Etemadi, Christian C. Abnet, Farhad Islami, Ramin Shakeri, Reza Malekzadeh, Sanford M. Dawsey

**Affiliations:** 1 Department of Anthropology, University of Texas at San Antonio, One UTSA Circle, San Antonio, TX, 78249, United States of America; 2 Division of Cancer Epidemiology and Genetics, National Cancer Institute, National Institutes of Health, 9609 Medical Center Dr., Bethesda, MD, 20892, United States of America; 3 Digestive Oncology Research Center, Digestive Disease Research Institute, Tehran University of Medical Sciences, Tehran, Iran; 4 Surveillance and Health Services Research, American Cancer Society, 250 Williams St., Atlanta, GA, 30303, United States of America; Peking University Cancer Hospital and Institute, CHINA

## Abstract

**Background:**

Gastric cancer (GC) is the world’s fifth most common cancer, and the third leading cause of cancer-related death. Over 70% of incident cases and deaths occur in developing countries. We explored whether disparities in access to improved drinking water sources were associated with GC risk in the Golestan Gastric Cancer Case Control Study.

**Methods and Findings:**

306 cases and 605 controls were matched on age, gender, and place of residence. We conducted unconditional logistic regression to calculate odds ratios (ORs) and 95% confidence intervals (CI), adjusted for age, gender, ethnicity, marital status, education, head of household education, place of birth and residence, homeownership, home size, wealth score, vegetable consumption, and *H*. *pylori* seropositivity. Fully-adjusted ORs were 0.23 (95% CI: 0.05–1.04) for chlorinated well water, 4.58 (95% CI: 2.07–10.16) for unchlorinated well water, 4.26 (95% CI: 1.81–10.04) for surface water, 1.11 (95% CI: 0.61–2.03) for water from cisterns, and 1.79 (95% CI: 1.20–2.69) for all unpiped sources, compared to in-home piped water. Comparing unchlorinated water to chlorinated water, we found over a two-fold increased GC risk (OR 2.37, 95% CI: 1.56–3.61).

**Conclusions:**

Unpiped and unchlorinated drinking water sources, particularly wells and surface water, were significantly associated with the risk of GC.

## Introduction

Gastric cancer (GC) is the world's fifth most common cancer, and the third leading cause of cancer-related death [1]. In many developed countries, the incidence of GC has declined dramatically. The primary factor behind the decline of GC incidence is the reduction of chronic *Helicobacter pylori* (*H*. *pylori)* infection [2], which is thought to be due to better personal hygiene and food preparation related to improved water sources and sanitary facilities [3]. Other factors attributed to the decline in GC include refrigeration, improved nutritional status, and decreased consumption of salted and preserved foods [2].

However, GC remains a significant burden in developing countries, where over 70% of incident cases and deaths occur [1,2]. Ecological data suggest that historical changes in international GC mortality correlate with infant mortality rates, a proxy for measuring impoverished living conditions [4]. Further, variation in the economic and geographic distribution of GC correlates with variation in the prevalence of *H*. *pylori* infection [3,5,6], the most important known risk factor for GC. This indicates that environmental factors might play a significant role in the incidence and mortality from GC [7,8].

Previous studies have found associations between water sources and the risks of GC [9–12], as well as between water sources and *H*. *pylori* infection [13]. Thus, one possible factor behind the observed geographic and economic disparities in GC rates are differences in improved drinking water sources that enable access to safe drinking water, defined by the World Health Organization as treated water of a standardized quality. Improved drinking water sources include in-home piped water, boreholes, protected dug wells and springs, rainwater, and public standpipes [14]. Of these, in-home piped water is associated with the best health outcomes [15,16].

As of 2010, an estimated 780 million people worldwide did not have adequate access to treated water, owing in part to a dearth of safe water infrastructure in many low-income countries, poverty, and disparities between urban and rural safe water infrastructure and in-home piped water coverage [16]. Even where infrastructure exists, access may be limited to only a few hours a day and quality may be poor [17].The acute health affects of inadequate access to safe drinking water, particularly diarrheal disease among children, are well documented. Less recognized are the longer-term health effects, including cancer [18].

The purpose of this study was to investigate whether GC and *H*. *pylori* seropositivity are associated with primary drinking water source in data from a case-control study of GC in northern Iran. Gastric cancer is the most common form of cancer in northern Iran, including high rates in Golestan Province [19]. Golestan, located in northeastern Iran, has experienced a relatively recent and rapid development of improved drinking water infrastructure. Yet inequalities in safe water access remain, particularly between urban and rural communities [16,20].

## Methods

### Study Design and Population

The Gastric and Esophageal Malignancies in Northern Iran (GEMINI) Phase I: Case-Control Study (henceforth referred to as the Golestan Case-Control Study of Gastric Cancer) has previously been described [7]. Briefly, histopathologically proven cases of gastric adenocarcinoma (GCA) were enrolled between December 2004 and December 2011 at Atrak Clinic, the only referral clinic for gastrointestinal diseases in Gonbad City, the largest city in eastern Golestan Province. Cases were enrolled from among patients with suspected upper gastrointestinal (GI) tract diseases who received upper GI endoscopy at Atrak Clinic. Pathologists at the Digestive Disease Research Center at Tehran University of Medical Sciences reviewed the biopsy samples, and those patients with adenocarcinoma of the stomach were invited to participate in the study. Written informed consent was obtained from each participant. The Institutional Review Boards of the U.S. National Cancer Institute, the Digestive Disease Research Center of Tehran University of Medical Sciences, and International Agency for Research on Cancer (IARC) reviewed and approved the study protocol. Additional information can be found at ClinicalTrials.gov using identifier NCT00339742.

Controls were selected from healthy subjects (n = 50,045) between the ages of 40 and 75 years who were enrolled in the Golestan Cohort Study between January 2004 and June 2008. For each case, two controls were individually matched to cases, where possible, on age, sex and neighborhood [21]. As described previously [7], most of the cases (83.4%) had two controls. However, we were unable to match two controls to all cases because controls were selected from the cohort study participants, who were limited to certain regions of the cases’ catchment area and were 40–75 years old at time of enrollment. Therefore, 6.6% of the cases had only one control. In addition, since both of the controls selected for 11 of the cases lacked adequate plasma samples, an additional (third) control was assigned to them

After dropping 165 cases and controls for which we had no water source data, our final study sample size (N = 911) consisted of 306 cases and 605 controls (Table 1). The Institutional Review Boards of the Digestive Disease Research Center of Tehran University of Medical Sciences, the US National Cancer Institute (NCI), and the International Agency for Research on Cancer (IARC) reviewed and approved the study protocol. Study participants gave written informed consent prior to participation. The authors have declared that no competing interests exist.

**Table 1:** Characteristics of 306 cases and 605 controls in the Golestan Gastric Cancer Case-Control Study.

	Controls	Cases	p-value*
N	605	306	
Age (years), mean (SD) ^#^	63.6 (9.2)	65.2 (10.8)	0.02
Female gender, N (%)^#^	167 (27.6)	82 (26.8)	0.80
Ethnicity, N (%)			0.00
Turkmen	368 (60.8)	145 (47.4)	
Fars	85 (14.1)	62 (19.6)	
Turk	74 (12.2)	47 (15.4)	
Sistani	56 (9.3)	32 (10.5)	
Others	22 (3.6)	22 (7.2)	
Married, N (%)	521 (86.1)	237 (77.5)	0.01
Education, N (%)			
No formal education	443 (73.2)	252 (82.4)	0.01
Head of household education, N (%)			
No formal education	371 (61.3)	214 (69.9)	0.00
Urban place of birth, N (%)	86 (14.2)	34 (11.4)	0.27
Urban residence, N (%)^#^	191 (31.6)	99 (32.5)	0.79
Home ownership, N (%)	586 (96.9)	284 (92.8)	0.01
Home size (m^2^), mean (SD)	106.0 (51.8)	97.7 (47.8)	0.02
Wealth score			0.14
First quintile	140 (23.1)	86 (28.4)	
Second quintile	119 (19.7)	50 (16.5)	
Third quintile	108 (17.9)	44 (14.52)	
Fourth quintile	120 (19.8)	72 (23.8)	
Fifth quintile	118 (19.5	51 (16.8)	
Total vegetable consumption (g/day), mean (SD)	185.7 (99.8)	176.5 (103.0)	0.21
*H*. *pylori* seropositivity, N (%)	441 (83.7)	233 (84.1)	0.87

Note: *H*. *pylori* infection data missing for 78 controls and 27 cases.

*P-values from Student’s *t* test (continuous variables) or Pearson’s chi squared test (categorical variables).

^#^ Matching variables

### Data Sources

Trained interviewers used structured questionnaires to collect data on age, sex, ethnicity, place of birth (urban or rural), place of current residence (urban or rural), personal history of disease (including cancer), tobacco and opiate use, alcohol and drug use, and other potential confounders of interest. Subjects were asked about their current primary drinking water sources and those of their prior residence. Primary drinking water sources were categorized as: piped water, chlorinated water, unchlorinated well, surface water, cistern water, or other. In the case of two sources, subjects were asked which source they used most often. In addition to analyzing individual sources, we grouped sources by whether they were piped into the home (referred to as “piped water”) or not piped into the home (referred to as “unpiped”). We then grouped sources by whether they were treated by chlorination into chlorinated (piped water and chlorinated well water) and unchlorinated (all other sources). Table 2 shows how each water source category was defined.

**Table 2:** Water Source Definitions.

Drinking Water Source		Controls, N (%)	Cases, N (%)	Total, N (%)
***Chlorinated***	*Piped water*: chlorinated water from a centralized water treatment source, including some wells, that is piped into the home.	511 (84.5)	246 (80.4)	757 (83.1)
	*Chlorinated well water*: water from a non-centralized well that has been chlorinated either by the household or by community health workers called *behvarz*, who are responsible for community safe water and water quality testing in rural areas [49].	27 (4.5)	2 (0.7)	29 (3.2)
***Unchlorinated***	*Unchlorinated well water*: water obtained from an unchlorinated well that is not accessed through a pipe leading into the home. Wells can be personal sources owned by one household, or they can be shared.	11 (1.8)	22 (7.2)	33 (3.6)
	*Surface water*: water retrieved from rivers, lakes, springs, or other natural sources that is not accessed through a pipe leading into the home.	10 (1.7)	16 (5.2)	26 (2.9)
	*Cistern water*: rain water collected and stored in underground reservoirs which have little ventilation or exposure to outside contamination. Cisterns are maintained and owned by households, and are not shared sources. This water is not accessed through a pipe leading into the home.	46 (7.6)	19 (6.2)	65 (7.1)

We collected 10 ml of venous blood, and stored 5 ml in EDTA anticoagulant as whole blood at -80°C. The other 5 ml without anticoagulant was centrifuged and the serum was stored at -80°C. A 10 ml sample of venus blood was also collected from controls, which was centrifuged and aliquoted into 500 μl straws (8 of plasma, 4 of buffy coat, and 2 of red blood cells). Case serum samples and control plasma samples were used to determine *H*. *pylori* seropositivity. Seropositivity to *H*. *pylori* was defined using a multiplex assay to 15 specific *H*. *pylori* antigens, as previously described [22,23]. We defined *H*. *pylori* positivity as those seropositive for ≥4 antigens, as in previously published studies [22–24].

### Statistical Analysis

Statistical analyses were performed using STATA version 11 (Stata Corp., College Station, TX)) and all *p*-values were two-sided. The distribution of baseline characteristics between cases and controls were compared using the Student’s *t* test for continuous variables and the Pearson’s chi squared test for categorical variables. Factors associated with water source were determined based on data from controls only, using the same statistical tests. We explored whether the following variables were associated with drinking water source in controls: age, gender, ethnicity, marital status, education, head of household education, urban or rural place of birth and residence, homeownership, size of home, wealth score, vegetable consumption, and *H*. *pylori* seropositivity. Of these, we found that only ethnicity, urban or rural place of birth, and urban or rural residence were significantly associated (p<0.05) with the type of drinking water source, and we adjusted for these variables in our final analyses. We also tried stratified analyses (by ethnicity, residence, place of birth and education), which were uninformative due to exposure homogeneity in some of the strata, thus we do not report those results here.

Using unconditional logistic regression, we calculated odds ratios (OR) and 95% confidence intervals (95% CI) for the association between gastric adenocarcinoma and current primary drinking water source. Fully-adjusted models included age and known risk factors for GC (gender, total vegetable consumption, and *H*. *pylori* seropositivity) as well as any variable which altered the β-estimate by more than 5% in a univariate model: ethnicity, marital status, urban or rural place of birth, and current urban or rural residence. In addition, we adjusted for education, home ownership, size of home, and household wealth score, as indicators of socio-economic status.

The wealth score was previously created using multiple correspondence analysis [25] as a composite score reflecting living conditions and household assets. Alcohol and smoking have previously been shown not to be risk factors for upper gastrointestinal cancers in this population [26,27], so we did not adjust for those variables. Because of the high prevalence of *H*. *pylori* infection in this population, we were not able to stratify our analysis by infection status. However, we conducted a comparative analysis among those who were *H*. *pylori* positive, and the results were similar to the larger population of both positive and negative subjects.

In addition to all gastric adenocarcinomas, we conducted subgroup analyses for cardia and noncardia gastric adenocarcinomas (GNCA), the two main anatomic subtypes. Previous studies have demonstrated that cardia and noncardia cancers may have different risk factors [28–30].

## Results

Table 1 shows the baseline characteristics of the 306 cases of gastric adenocarcinoma (161 cardia, 115 non-cardia, and 30 with mixed or unspecified site) and 605 matched controls. Cases and controls were very closely matched on gender (27% of cases and 28% of controls were female) and urban/rural residence (33% of cases and 32% of controls lived in urban areas), two of the matching variables. There was a small but significant difference in the third matching variable, age: the mean age of cases (65.2 years) exceeded that of controls (63.6 years) by 1.6 years (p = 0.02). Among the other variables, cases were less likely than controls to be of Turkmen ethnicity, married, have formal education, have a head of household who had formal education, or own a home. Both cases and controls were more likely to have been born and currently live in rural areas. Neither the distribution of wealth nor mean daily vegetable consumption was significantly different between cases and controls. Both groups had high rates of *H*. *pylori* seropositivity.

Table 3 shows the factors associated with type of current drinking water source, based on data from controls only. Significant associations were found between current source of water and ethnicity, urban or rural place of birth, urban or rural place of residence, wealth score, and daily vegetable consumption. *H*. *pylori* seropositivity was not associated with current drinking water source.

**Table 3:** Factors associated with drinking water source among controls in the Golestan Gastric Cancer Case-Control Study.

Current water source	Chlorinated	Chlorinated	Unchlorinated	Unchlorinated	Unchlorinated	
	Piped water (N = 511, 84.5%)	Well (N = 27, 4.5%)	Well (N = 11, 1.8%)	Surface (N = 10,1.7%)	Cistern (N = 46,7.6%)	p-value*
Age (years), mean (SD)	63.7 (9.4)	63.7 (6.4)	59.8 (6.8)	65.7 (9.5)	63.1 (9.02)	0.64
Gender, N (%)						0.58
Female	144 (86.2)	9 (5.4)	2 (1.2)	1 (0.6)	11 (6.6)	
Male	367 (83.8)	18 (4.1)	9 (2.1)	9 (2.1)	35 (8.0)	
Ethnicity, N (row %)						0.00
Turkmen	280 (76.1)	27 (7.3)	5 (1.4)	10 (2.7)	46 (12.5)	
Other	231 (97.5)	0 (0.0)	6 (2.5)	0 (0.0)	0 (0.0)	
Marital status, N (%)						0.95
single	3 (100.0)	0 (0.0)	0 (0.0)	0 (0.0)	0 (0.0)	
married	442 (84.8)	19 (3.7)	10 (1.9)	9 (1.7)	41 (7.9)	
widow	64 (81.0)	8 (10.1)	1 (1.3)	1 (1.3)	5 (6.3)	
divorced	1 (100.0)	0 (0.0)	0 (0.0)	0 (0.0)	0 (0.0)	
Education (N, %)						0.14
No formal education	370 (83.5)	22 (5.0)	6 (1.4)	10 (2.3)	35 (7.9)	
Some education	141 (87.0)	5 (3.1)	5(3.1)	0 (0.0)	11 (6.8)	
Head of household education (N, %)						0.12
No formal education	310 (83.6)	15 (4.0)	6 (1.6)	10 (2.7)	30 (8.1)	
Some education	201(85.9)	12 (5.1)	5 (2.1)	0 (0.0)	16 (6.8)	
Place of birth (N, %)						0.01
Rural	426 (82.2)	27 (5.2)	10 (1.9)	10 (1.9)	45 (8.7)	
Urban	84 (97.7)	0 (0.0)	1 (1.2)	0 (0.0)	1 (1.2)	
Place of residence (N, %)						0.00
Rural	321 (77.5)	27 (6.5)	11 (2.6)	10 (2.4)	45 (10.9)	
Urban	190 (99.5)	0 (0.0)	0 (0.0)	0 (0.0)	1 (0.5)	
Home ownership (N, %)	495 (84.5)	27 (4.6)	9 (1.5)	10 (1.7)	45 (7.7)	0.08
Home size, mean (m^2^) (SD)	108.2 (54.8)	88.3 (34.1)	90.4 (25.1)	83.5 (22.6)	100.3 (25.7)	0.11
Wealth score						0.00
First quintile	108 (77.1)	14 (10.0)	6 (4.3)	4 (2.9)	8 (5.7)	
Second quintile	99 (83.2)	6 (5.0)	0 (0.0)	6 (5.0)	8 (6.7)	
Third quintile	94 (87.0)	2 (1.9)	4 (3.7)	0 (0.0)	8 (7.4)	
Fourth quintile	106 (88.3)	4 (3.3)	0 (0.0)	0 (0.0)	10 (8.3)	
Fifth quintile	104 (88.1)	1 (0.9)	1 (0.9)	0 (0.0)	12 (10.2)	
Total vegetable consumption (g/day), mean (SD)	190.9 (102.5)	145.5 (88.3)	186.1 (62.0)	150.4 (74.7)	160.7 (77.5)	0.04
*H*. *pylori* seropositivity (N, %)	390 (84.4)	5 (71.4)	8 (88.9)	7 (77.8)	31 (77.5)	0.65

Note: *H*. *pylori* infection data was missing for 78 controls.

*P-values from Student’s *t* test (continuous variables) or Pearson’s chi squared test (categorical variables).

To explore how the risk of GC differed by primary drinking water source, we first explored the risk of GC by individual water sources. As shown in Table 4, unadjusted models showed that, compared to in-home piped water, those who reported primarily drinking chlorinated well water (n = 2) had an 84% reduction in risk (OR 0.16; 95% CI: 0.04–0.66). In contrast, we found an increased risk of GC among those whose current primary drinking water source was unchlorinated well (OR 4.20, 95% CI: 2.01–8.81) or surface water (OR 3.36; 95% CI: 1.50–7.52). The associations for unchlorinated well and surface water were maintained in the multivariate models that were fully adjusted for age, gender, ethnicity, marital status, education, head of household education, urban or rural place of birth and residence, homeownership, size of home, wealth score, vegetable consumption, and *H*. *pylori* seropositivity. Multivariate models showed ORs of 4.58 (95% CI: 2.07–10.16) for unchlorinated well water, and 4.26 (95% CI: 1.81–10.04) for surface water, after adjusting for the aforementioned variables. The OR for chlorinated well water (OR 0.23, 95% CI: 0.05–1.04) was no longer significant after full adjustment. No significant associations were found for cistern water.

**Table 4:** Odds Ratios and 95% CIs for Gastric Cancer (GC) by Water Source.

			OR (95% CI)		
	Controls, N	Cases, N	Unadjusted	Age-adjusted	Fully adjusted model, *
**Individual Water Sources**					
Piped water	511	246	1.00 (ref)	1.00 (ref)	1.00 (ref)
Chlorinated well	27	2	0.16 (0.04–0.66)	0.16 (0.04–0.66)	0.23 (0.05–1.04)
Unchlorinated well	11	22	4.20 (2.01–8.81)	4.55 (2.16–9.60)	4.58 (2.07–10.16)
Surface water	10	16	3.36 (1.50–7.52)	3.33 (1.48–7.46)	4.26 (1.81–10.04)
Cistern	46	19	0.89 (0.50–1.51)	0.88 (0.50–1.53)	1.11 (0.61–2.03)
**Unpiped Versus Piped Water Sources**					
Piped	511	246	1.00 (ref)	1.00 (ref)	1.00 (ref)
Unpiped	94	60	1.33 (0.93–1.90)	1.35 (0.94–1.93)	1.79 (1.20–2.69)
**Unchlorinated versus Chlorinated Drinking Water Sources**					
Chlorinated	538	248	1.00 (ref)	1.00 (ref)	1.00 (ref)
Unchlorinated	67	58	1.88 (1.28–2.75)	1.92 (1.31–2.82)	2.37 (1.56–3.61)

*Adjusted for age, gender, ethnicity, marital status, education, head of household education, urban or rural place of birth and residence, home ownership, size of home, wealth score, vegetable consumption, and *H*. *pylori* seropositivity.

Next, we combined sources and investigated the risk associated with unpiped sources (n = 60 cases) compared to sources piped into the home (n = 246 cases). Unadjusted models shown in Table 4 indicated a 33% increased risk of GC (OR 1.33, 95% CI: 0.93–1.90) for those drinking from unpiped water sources compared to in-home piped ones. After full adjustment, we found that those drinking from unpiped sources had a 79% higher risk of GC than those drinking water piped into their homes (OR 1.79, 95% CI: 1.20–2.69).

We then explored the difference in risk between chlorinated (piped and chlorinated well water; n = 248 cases) and unchlorinated (unchlorinated well, surface water, and cistern water; n = 58 cases) drinking water sources. In unadjusted models, we found an 88% increased risk of GC (OR 1.88, 95% CI: 1.28–2.75) for those drinking from unchlorinated water sources compared to chlorinated ones (Table 4). Fully-adjusted models indicated that those drinking from unchlorinated sources had over twice the risk of those drinking chlorinated water (OR 2.37, 95% CI: 1.56–3.61).

We also analyzed the association of water source and the risk of GC separately for cardia (GCA) and noncardia adenocarcinomas (GNCA) (Table 5). Fully-adjusted models indicated that the magnitude of association for unchlorinated well water was larger for GCA (OR 7.17, 95% CI: 2.96–17.35) than for GNCA (OR 2.45, 95% CI: 0.76–7.92), but these results were based on small numbers (15 GCA cases, 6 GNCA cases, 11 controls). For surface water, we found a higher risk for GNCA (OR 5.53, 95% CI: 1.92–15.99) than for GCA (OR 3.03, 95% CI: 1.03–8.93), also based on small numbers (7 GCA, 8 GNCA, 10 controls). The associations between unpiped water and unchlorinated water and anatomic subtypes were similar to the associations for all GCs.

**Table 5:** Odds Ratios and 95% CIs for GC, GCA, and GNCA by water sources.

		All GC^#^		GCA		GNCA	
	Controls, N	Cases, N	OR (95% CI)*	Cases, N	OR (95% CI) *	Cases, N	OR (95% CI) *
**Individual water source**							
Piped water	511	246	1.00 (ref)	127	1.00 (ref)	92	1.00 (ref)
Chlorinated well	27	2	0.23 (0.05–1.04)	1	0.22 (0.03–1.81)	1	0.32 (0.04–2.72)
Unchlorinated well	11	22	4.58 (2.07–10.16)	15	7.17 (2.96–17.35)	6	2.45 (0.76–7.92)
Surface water	10	16	4.26 (1.81–10.04)	7	3.03 (1.03–8.93)	8	5.53 (1.92–15.99)
Cistern	46	19	1.11 (0.61–2.03)	11	1.08 (0.51–2.28)	7	1.26 (0.50–3.15)
**Unpiped sources**							
Piped	511	246	1.00 (ref)	127	1.00 (ref)	92	1.00 (ref)
Unpiped	94	60	1.79 (1.20–2.69)	34	1.93 (1.17–3.20)	23	1.97 (1.09–3.56)
**Unchlorinated sources**							
Chlorinated	538	248	1.00 (ref)	128	1.00 (ref)	93	1.00 (ref)
Unchlorinated	67	58	2.37 (1.56–3.61)	33	2.53 (1.52–4.22)	22	2.52 (1.38–4.61)

^#^All GC includes GCA (n = 127), GNCA (n = 92), and 30 mixed or unspecified sites.

*Adjusted for age, gender, ethnicity, marital status, education, head of household education, urban or rural place of birth and residence, home ownership, size of home, wealth score, vegetable consumption, and *H*. *pylori* seropositivity.

Finally, we explored whether the duration of unpiped water use might be associated with the risk of GC (Table 6). Although the difference in the odds of GC by quartiles of unpiped water use duration were not statistically significant (p trend = 0.06), it seemed that among people who had used unpiped water for more than 53 years, the odds of having gastric cancer were higher compared with those who used it for less than 30 years (OR 1.48; 95%CI: 0.87–2.53). Analysis of this risk by anatomic subsite was insignificant.

**Table 6:** Odds Ratios and 95% CIs for Gastric Cancer by Duration Unpiped Water Source.

	OR (95% CI)		
Duration unpiped (years, # cases)	Unadjusted	Age-adjusted	Fully-adjusted model*
First quartile (0–31 years, n = 72)	1.00 (ref)	1.00 (ref)	1.00 (ref)
Second quartile (32–42 years, n = 70)	0.98 (0.66–1.46)	0.88 (0.58–1.33)	0.86 (0.54–1.38)
Third quartile (43–52 years, n = 68)	1.02 (0.68–1.51)	0.89 (0.58–1.36)	0.85 (0.51–1.41)
Fourth quartile (53+ years, n = 94)	1.44 (0.99–2.11)	1.21 (0.79–1.86)	1.48 (0.87–2.53)

*Adjusted for age, gender, ethnicity, marital status, education, head of household education, urban or rural place of birth and residence, home ownership, size of home, wealth score, vegetable consumption, and *H*. *pylori* seropositivity.

## Discussion

Our study demonstrates a strong association between the primary use of unpiped and unchlorinated drinking water sources, specifically unchlorinated well and surface water, and an increased risk of gastric adenocarcinoma, both GCA and GNCA. After fully adjusting for potential confounders, including age, gender, ethnicity, marital status, education, head of household education, urban or rural place of birth and residence, home ownership, size of home, wealth score, vegetable consumption, and *H*. *pylori* seropositivity, we found an over 4-fold increased risk for drinking unchlorinated well or surface water compared to piped water. In addition, we found a 79% increased risk of GC among those whose primary drinking water came from unpiped sources versus in-home piped ones. Further, our analysis comparing water that was treated through chlorination (both piped and well water) to untreated sources indicated that those using unchlorinated drinking water sources had over twice the risk of developing GC.

Our finding that drinking water source influences the risk of GC is consistent with the limited number of studies that have evaluated this association. Well water in particular has been found in other regions to be associated with a higher risk of GC. Based on 783 GC cases and 1,566 hospital controls in Japan, Haenzel and colleagues [10] found an OR of 1.5 (p-value<0.01) for those drinking well water compared to those drinking water from public sources, and an even higher risk (OR 1.9; p-value<0.01) among the well water drinkers living on farms. Boeing and colleagues [12] found a relative risk of 2.26 (95% CI 1.19–4.28) among well water users in Germany compared to those with centralized water sources, based on 21 GC cases and 55 controls. A higher risk of GC precursor lesions has also been associated with well water use [11]. Public and in-home piped drinking water sources have also been found to be protective. In Canada, Risch and colleagues found a 14% reduction in GC risk among those who had used public water supplies for 10 years compared to non-public ones (OR for ten year exposure: 0.86, 95% CI: 0.76–0.99) [31]. More recently, a study conducted in Linxian, China, found that in-home piped water was protective against GCA (OR 0.81, 95% CI: 0.70–0.94), but found no significant association with GNCA (OR 0.99, 95% CI: 0.78–1.26) [32].

Our risk estimate for drinking unchlorinated well water is 2–3 times higher than these previous estimates, and our results show a greater protective effect for in-home piped (i.e. public) drinking water. In addition, our subsite analysis found a significant inverse association of greater magnitude for in-home piped water and both GCA and GNCA.

One possible mechanism that has been suggested for an association between primary water source and GC risk is water quality, specifically variation in levels of nitrites, nitrates, calcium, magnesium, and other constituents that might increase the risk of GC [9,31,33]. Although the present study does not include data on water quality, another study in Golestan [20] found that higher concentrations of nitrates in drinking water sources correlated with areas of higher risk for esophageal cancer. However, studies have reported mixed results regarding possible associations between water nitrate levels and GC risk [34,35].

A second possible mechanism is contamination of unimproved water sources by the bacterium *H*. *pylori*. However, in our data, we did not see an association between *H*. *pylori* seropositivity and GC risk, owing to the high prevalence of infection throughout this population. In a population with lower *H*. *pylori* prevalence, it is possible that higher risks of GC among those using unchlorinated well and surface water might be explained by a higher risk of *H*. *pylori* infection among those with unimproved drinking water sources. *H*. *pylori* infection has been found to be associated with a lack of in-home piped water supply [36–38], a lack of a fixed hot water supply [39], use of wells [40], drinking water collected from streams [41], use of cisterns, and use of a community water tap drawn from surface water contaminated by industrial and domestic fecal pollution [36]. Untreated water is also associated with a higher prevalence of *H*. *pylori* [42], while chlorinated water has been found to lack the bacterium [43]. Water source might also contribute to *H*. *pylori* infection through the amount of water accessible for hygiene, as poor hygiene is associated with a higher seroprevalence of *H*. *pylori* infection, especially during childhood [3,38,44]. Living conditions such as lack of a bathroom or hot water supply, and household crowding with very limited sanitation facilities are also associated with *H*. *pylori* infection [3], while in-home piped water decreases the risk of infection by increasing access to clean water for both drinking and domestic uses (including preparation of raw vegetables) [45]. Finally, unchlorinated water sources can also expose individuals to microbes other than *H*. *pylori*. Colonization of the stomach by non-*H*. *pylori* bacteria may have a role in GC pathogenesis, especially in people with atrophic gastritis and decreased acid secretion [46].The strengths of this study include a relatively large sample size, the use of previously validated and reliable questionnaires, histologic diagnosis of all adenocarcinoma cases, and use of population-based controls that have been previously demonstrated to be representative of the population as a whole [7,27].

One limitation of this analysis is that the study was not designed to evaluate water source as a risk factor for GC, and the sample size of water sources (other than piped) are small. Further, we did not have data on drinking water quality, whether that quality differed in urban or rural settings, how people accessed their primary water source if it was not a piped source, or the number and quantity of other sources used. There was also a high prevalence of *H*. *pylori* seropositivity in the region, so our ability to study the effect of this infection was limited.

Finally, our study did not collect data on daily sodium intake, which has been demonstrated in other studies to increase the risk of GC [47], and to increase the positive association of *H*. *pylori* and GC risk [48]. This probably has little impact on our results for two reasons: first, most of the associations between salt intake and gastric cancer have been observed in studies from Japan, and not replicated in other populations; second, dietary salt intake is unlikely to be associated with drinking water source.

In conclusion, we found a strong association between drinking unchlorinated water from wells and surface water sources and GC risk. This association remained significant after adjustment for education, home ownership, home size, and wealth score, demonstrating that this higher risk is separate from individual indicators of socioeconomic status. Further studies on the quality, mechanisms of access, and rates of consumption of specific water sources are needed to explain this association. Our findings suggest the importance of considering the role of improved water sources in the prevention of GC. This may be particularly significant for reducing the burden of GC in areas characterized by poor improved water source coverage. Further, our findings add to the growing recognition of the breadth of disease burden associated with inequalities to safe drinking water access.
